# Comparative effect of statins and types of physical exercise on arterial stiffness: Protocol for network meta-analysis

**DOI:** 10.1097/MD.0000000000015484

**Published:** 2019-05-17

**Authors:** Vicente Martínez-Vizcaíno, Iván Cavero-Redondo, Alicia Saz-Lara, Diana P. Pozuelo-Carrascosa, Blanca Notario-Pacheco, Manuel A. Gómez-Marcos, Celia Álvarez-Bueno

**Affiliations:** a Universidad de Castilla-La Mancha, Health and Social Research Center, Cuenca, Spain; b Universidad Autónoma de Chile, Facultad de Ciencias de la Salud, Talca, Chile; c Institute of Biomedical Research of Salamanca (IBSAL), Primary Health Care Research Unit, La Alamedilla Health Center. Health Service of Castilla y León (SACYL), Primary Care Prevention and Health Promotion Research Network (REDIAPP); d Department of Medicine, University of Salamanca, Salamanca, Spain.

**Keywords:** arterial stiffness, network meta-analysis, physical exercise, pulse wave velocity, statins

## Abstract

**Introduction:**

The purpose of this study protocol is to provide the methodology for a review to compare the effect of statins vs physical exercise interventions and the effect of different types of physical exercise, on reducing arterial stiffness associated with cardiovascular diseases and mortality.

**Methods and analysis:**

The literature search will be conducted in MEDLINE, EMBASE, Cochrane Central Register of Controlled Trials, Cochrane Database of Systematic Reviews, and Web of Science databases from their inception until July 31, 2019. We will include randomized controlled trials, nonrandomized experimental studies, and controlled pre–post studies assessing the effect in the general population of statins and physical exercise interventions on arterial stiffness measured by pulse wave velocity. The Cochrane Collaboration's tool and the Quality Assessment Tool for Quantitative Studies will be used to assess the risk of bias for studies included in the systematic review. A Bayesian network meta-analysis will be carried out to determine the comparative effect of the different physical exercise interventions and/or statin intervention.

**Ethics and dissemination:**

This study will generate evidence about the effectiveness of both statins and exercise on reducing arterial stiffness that potentially can be transferred to patients and practitioners. Moreover, in light of the importance of reducing arterial stiffness for preventing cardiovascular disease, the evidence provided by this study will be potentially suitable to be included in cardiovascular clinical practice guidelines.

**Strengths and limitations:**

This protocol describes the methods of a study examining, using network meta-analysis strategies, the efficacy of statins and different types of exercise on improving arterial stiffness, which is an early marker of atherosclerosis. The results of this study could immediately help clinicians to recommend the best evidence-based intervention to their patients to reduce arterial stiffness and, as a consequence, prevent major complications, such as heart failure, stroke, or myocardial infarction.

**Trial registration number:**

PROSPERO CRD42019123120

## 1 Introduction

Arterial stiffness is a marker of early stages of vascular aging^[1]^ and is significantly associated with an increased risk of cardiovascular events and mortality.^[2]^ Inflammation and oxidative stress have been proposed as the underlying mechanisms responsible for the stiffening of vessels’ walls,^[3]^ as they are responsible for elastin fragmentation, collagen deposition, and smooth muscle cell proliferation.^[4]^ During the last 2 decades, there has been accumulating evidence supporting the hypothesis that arterial stiffness explains a significant percentage of cardiovascular risk that cannot be explained by hypertension, dyslipidemia, smoking, or physical inactivity, and the evidence advocates the inclusion of arterial stiffness measurements in the assessment of cardiovascular risk in clinical practice.^[5]^

The accepted gold standard for noninvasive measurement is pulse wave velocity (PWV), as it has proved to be an independent predictor of cardiovascular events.^[6]^ The beneficial role of both exercise and statin therapy on arterial stiffness has been repeatedly reported. Additionally, experimental studies have reported that statin therapy, in addition to its consistently favorable effect on the lipid profile, reduces cardiovascular risk by several mechanisms, such as improving nitric oxide bioavailability,^[7]^ their antioxidant effect,^[8]^ or their influence on the renin–angiotensin–aldosterone system.^[9]^

The physiological mechanisms by which physical exercise benefits arterial stiffness have not been completely established. Exercise induces the relaxation of vascular smooth muscle cells by increasing arterial wall shear stress and nitric oxide activity, as well as reducing oxidative stress, inflammation, and vasodilation.^[10]^ Although these effects are independent of the influences of exercise on traditional risk factors, the concurrent or mediating blood pressure-lowering effect might foster the effect of reducing stiffness.^[11]^

Thus, both statin therapy and physical exercise have been shown to be effective, nevertheless, the former has prevailed over the latter, and the costs associated with pharmacological treatment are high and the overall results suboptimal. Therefore, the struggle to achieve significant reductions in the incidence of cardiovascular events in the general population has not diminished.^[12]^ The most used prevention strategies focus on easy access to pharmacological prevention for the majority of the population^[13]^ and on identifying patients who have a higher risk of developing cardiovascular events within 10 years, to focus preventive efforts on them.^[14]^ Currently, the latter strategy is the preferred choice due to its low cost and reduced likelihood of side effects from the drugs.

Previous systematic reviews and meta-analyses have analyzed the effect of statins^[15–17]^ and physical exercise^[18–20]^ on arterial stiffness, but none have performed a comparative analysis of the effect of statins and different types of physical exercise on arterial stiffness. Therefore, the purpose of this protocol is to provide the methodology for a review to compare the effect of statins vs physical exercise interventions, and the effect of different types of physical exercise, on reducing arterial stiffness associated with cardiovascular diseases (CVDs) and mortality.

## 2 Objectives

This network meta-analysis protocol presents an objective and clear procedure for the extraction of information from experimental studies (randomized controlled trials [RCTs], nonrandomized experimental studies, and controlled pre–post studies), in which data on changes in arterial stiffness measured by PWV are reported as the outcome to compare the dose response of statins and physical exercise on arterial stiffness and compare the effect of different types of physical exercise (continuous aerobic exercise, interval exercise training, strength, stretching, alternative exercise [Pilates, yoga, or Tai Chi], or a combination of exercises) on arterial stiffness.

## 3 Methods and analysis

### 3.1 Study registration

This network meta-analysis protocol is based on the Preferred Reporting Items for Systematic Review and Meta-Analysis Protocols^[21]^ and the Cochrane Collaboration Handbook.^[22]^ This protocol has been previously registered in the PROSPERO (registration number: CRD42019123120). As no primary data will be collected, approval from an ethics committee is not required.

### 3.2 Inclusion/exclusion criteria for study selection

#### 3.2.1 Type of studies

RCTs, nonrandomized experimental studies, and controlled pre–post studies will be included, without language restrictions.

#### 3.2.2 Type of participants

Studies assessing the effect in the general population of statins and physical exercise interventions on arterial stiffness measured by PWV will be selected. Studies will be selected regardless of the age of the participants included. When more than 1 study provides data referring to the same sample, we will choose the one presenting the most detailed results or providing the largest sample size.

#### 3.2.3 Type of interventions

Studies reporting any type of intervention consisting mainly of statin treatment or physical exercise (endurance, resistance, high interval training, stretching, or alternatives [Pilates, yoga, or Tai Chi]) understood as repeated bouts of physical exercise over time involving more than 1 sessions/week with a duration of at least 1 week, will be eligible for inclusion. Studies comparing statins and physical exercise interventions, comparing different types of physical exercise interventions, and examining statin treatment or a specific physical exercise intervention with or without a control group will be eligible for inclusion. However, studies combining statins or physical exercise with other health interventions, such as nutritional interventions, will be excluded when data concerning the effect of statins or physical exercise interventions on arterial stiffness cannot be extracted separately.

#### 3.2.4 Type of outcome assessment

Arterial stiffness parameters – several sites for PWV have been traditionally used for measuring arterial stiffness. Carotid-femoral PWV (cfPWV) measured by Doppler ultrasound is the most widely used measure of aortic stiffness and is recognized as the gold standard measure for evaluating arterial stiffness^[6]^; however, other additional PWV measurements sites, such as the brachial-ankle PWV (baPWV) and the cardiac-ankle PWV (caPWV),^[23]^ will be considered for inclusion in our systematic review.

### 3.3 Search methods for the identification of studies

#### 3.3.1 Electronic search

The literature search will be conducted in MEDLINE, EMBASE, Cochrane Central Register of Controlled Trials, Cochrane Database of Systematic Reviews, and Web of Science databases from the date of their inception until July 31, 2019. The searches will be repeated just before the final analyses to search for further potential studies. Study records will be managed using the Mendeley reference manager.

The following search terms will be combined by Boolean operators to conduct the literature search: “physical activity”, “physical fitness”, “physical exercise”, exercise, “intense exercise”, “exercise training”, “interval training”, “endurance training”, “resistance training”, “high interval training”, HIIT, “aerobic exercise”, stretching, strength, yoga, pilates, “Tai chi”, statins, atorvastatin, fluvastatin, lovastatin, pravastatin, rosuvastatin, simvastatin, “endothelial function”, “arterial stiffness”, “pulse wave velocity”, PWV, “randomized control trial”, RCT, “quasi-experimental study”, non-RCT, and “controlled pre-post study” (Table 1).

**Table 1:**
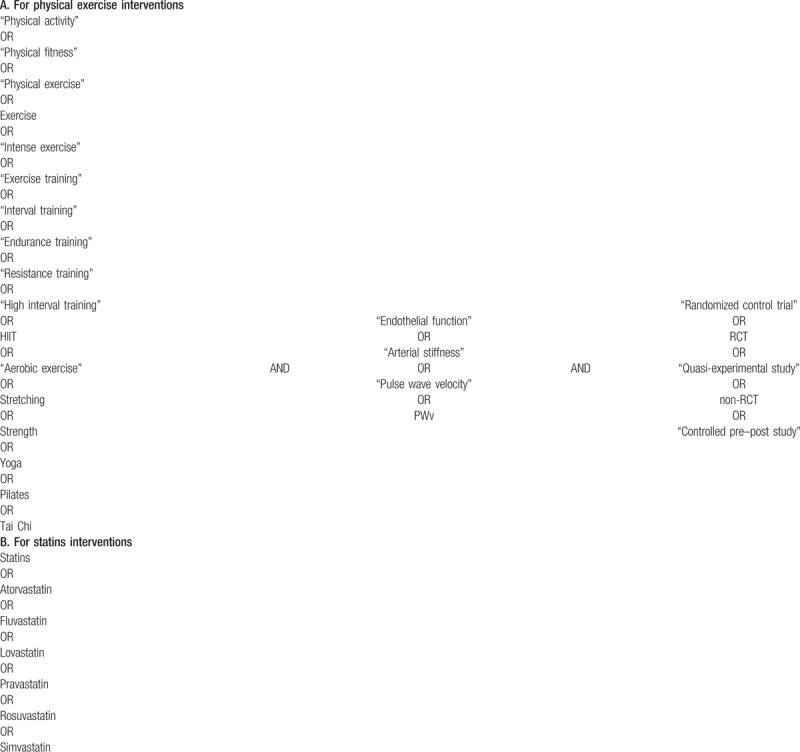
Search strategy for MEDLINE.

Previous reviews and meta-analyses and relevant references cited in the selected studies will be screened.

### 3.4 Data collection and analysis

#### 3.4.1 Selection of studies

**Figure 1:**
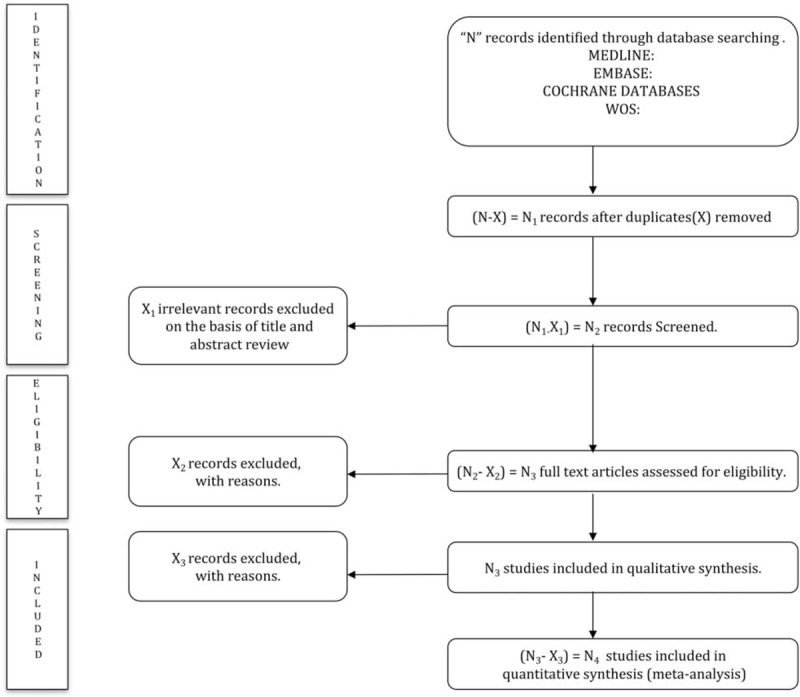
PRISMA flow diagram of identification, screening, eligibility, and inclusion of studies.

The title and abstract of retrieved articles will be independently evaluated by 2 reviewers to identify eligible studies according to the inclusion criteria. Then, the full manuscripts of the identified studies will be examined. Finally, the 2 reviewers will examine the included and excluded studies to verify the reasons for inclusion/exclusion (Fig. 1). Abstracts not providing enough information regarding the inclusion/exclusion criteria will be selected for full-text evaluation. The reviewers will not be blinded to the authors, institutions, or journals of the reviewed articles. Disagreements will be solved by consensus; when disagreements persist after discussion, a third reviewer will be required.

Two authors will independently extract information about the main study characteristics from the included studies, including authors, year of publication, country, study design, number and age of participants, population characteristics (healthy or with any specific disease), methods used for arterial stiffness measurement (cfPWV, baPWV, or caPWV), PWV mean values before the intervention, and type and characteristics of the intervention (Table 2).

**Table 2:**
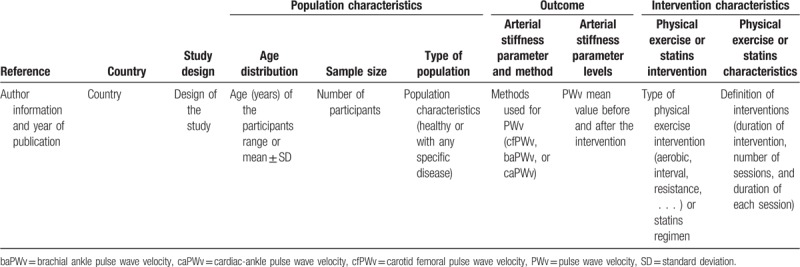
Characteristics of studies included in the systematic review and/or meta-analysis.

To avoid the double counting of patients that have been included in more than 1 report by the same author or working group, the recruitment periods will be evaluated. When necessary, corresponding authors of the potentially included studies will be contacted to obtain any missing information.

Any disagreements will be resolved by discussion to reach a consensus.

#### 3.4.2 Assessment of risk of bias in the included studies

Two researchers will independently conduct a quality assessment according to the Cochrane Collaboration Handbook recommendations.^[22]^ Any disagreements will be resolved by discussion and a third reviewer will solve disagreements if a consensus is not reached.

The methodological quality of the RCTs will be assessed using the Cochrane Collaboration's tool for assessing risk of bias (RoB2).^[24]^ This tool evaluates the risk of bias according to 6 domains: selection bias, performance bias, detection bias, attrition bias, reporting bias, and other bias.

The Quality Assessment Tool for Quantitative Studies^[25]^ will be used to assess the quality of pre–post studies and non-RCTs. This tool evaluates 7 domains: selection bias, study design, confounders, blinding, data collection method, withdrawals, and drop-outs.

For both quality assessment tools, each domain will be considered as strong, moderate, or weak, and studies will be classified as having a low risk of bias (with no weak ratings), a moderate risk of bias (with 1 weak rating), or a high risk of bias (with 2 or more weak ratings). The agreement rate between reviewers will be reported by calculating kappa statistics.

#### 3.4.3 Grading the quality of evidence

The Grading of Recommendations, Assessment, Development, and Evaluation (GRADE) tool will be used to evaluate the quality of the evidence and make recommendations.^[26]^ Each outcome will obtain a high, moderate, low, and very low evidence value, depending on the design of the studies, risk of bias, inconsistency, indirect evidence, imprecision, and publication bias.

## 4 Data synthesis

The included clinical trials will be summarized qualitatively in an ad hoc table describing the types of direct and indirect comparisons. The reviewers will determine whether a meta-analysis is possible after data extraction. Where a meta-analysis is not feasible, we will undertake a narrative synthesis. If a meta-analysis is possible, a standard meta-analysis for each direct comparison between 2 interventions will be performed using the random effects DerSimonian-Laird method^[27]^ and the statistical heterogeneity will be analyzed by calculation of the I^2^ statistic. According to the values of I^2^, the heterogeneity will be considered as not important (0% to 40%), moderate (30% to 60%), substantial (50% to 90%), or considerable (75% to 100%). Additionally, the corresponding *P* values will also be considered.

When it is possible to determine the comparative effect of the different physical exercise interventions and/or statin intervention, a Bayesian network meta-analysis will be carried out. The effects of each intervention will be combined using Bayesian methods of the Markov-Monte Carlo chain using STATA 15 (StataCorp, College Station, TX). The model developed by Dias *et al*.^[28]^ for the UK National Institute for Health and Care Excellence Decision Support Unit will be used.

The probability that each intervention, statin or physical activity, is the most effective will be presented graphically using rankograms. Additionally, the surface under the cumulative ranking (SUCRA) will be estimated for each intervention. SUCRA involves the assignment of a numerical value between 0 and 1 to simplify the classification of each intervention in the rankogram. The best intervention would obtain a value for SUCRA close to 1 and the worst intervention would be a value close to 0.^[29]^

## 5 Discussion

Over the last 30 years, cholesterol-lowering statins have become one of the most prescribed drugs worldwide, in part, because heart disease and stroke are among the world's leading causes of mortality.^[30]^ A Cochrane review showed that people with normal cholesterol levels should take statins when their risk of stroke or heart attack within 10 years is considered to be greater than 10%.^[31]^ In contrast, the threshold level for statin intervention recommended by the American Heart Association is 7.5%.^[32]^

However, the threshold for when to take statins is a complicated decision. For example, statins appear to vary in effectiveness depending on whether they are used as a primary or secondary prevention. Statins have a significant impact on preventing future heart failure (and mortality from CVD) in patients with previous heart disease (secondary prevention).^[33]^ However, the results are more variable in people without previous heart failure (primary prevention).^[34]^ Some previous reviews suggest that, for example, all-cause mortality does not change in people taking statins for primary prevention,^[35,36]^ although it should be noted that the most recent Cochrane review contradicts this conclusion.^[31]^

Another approach for the prevention of CVD is physical exercise, which is associated with a lower risk of CVD and mortality.^[37,38]^ However, it has not been established whether the effect of physical exercise on arterial stiffness is greater than statins, or which type of physical exercise is most effective at improving arterial stiffness. Instead, the combined effect of statins with physical exercise has been analyzed, noting that their combined effect substantially enhances the reduction in cardiovascular risk and mortality.^[39]^ Of note, these results are controversial due to the decreased ability to perform physical exercise as a result of the muscle pain caused by statins, which could eventually decrease the effect on endothelial function.^[40]^

Finally, this controversy between statins and physical exercise also illustrates the relationship between research and policy. Evidence-based policy attempts to address what is known, what is not known, and the challenges that remain unsolved. Therefore, policymakers should always try to incorporate these facets of uncertainty into the decision-making processes.

Thus, this protocol aims to provide a novel methodology for a network meta-analysis of the effect of statins and different types of physical exercise on arterial stiffness to prevent major complications, such as heart failure, stroke, or myocardial infarction. This study aims to generate evidence that can be applied in the decision making of policymakers and, therefore, to clinical practice guidelines to prescribe the best interventions to maintain optimal endothelial function.

## Author contributions

**Conceptualization:** Vicente Martínez-Vizcaíno, Iván Cavero-Redondo, Celia Álvarez-Bueno.

**Data curation:** Iván Cavero-Redondo, Alicia Saz-Lara.

**Formal analysis:** Iván Cavero-Redondo, Alicia Saz-Lara.

**Funding acquisition:** Vicente Martínez-Vizcaíno, Blanca Notario-Pacheco.

**Investigation:** Iván Cavero-Redondo.

**Methodology:** Iván Cavero-Redondo, Diana P. Pozuelo-Carrascosa, Manuel A. Gómez-Marcos, Celia Álvarez-Bueno.

**Supervision:** Vicente Martínez-Vizcaíno, Blanca Notario-Pacheco, Manuel A. Gómez-Marcos, Celia Álvarez-Bueno.

**Writing – original draft:** Iván Cavero-Redondo, Celia Álvarez-Bueno.

**Writing – review & editing:** Vicente Martínez-Vizcaíno, Diana P. Pozuelo-Carrascosa.

Iván Cavero-Redondo orcid: 0000-0003-2617-0430.
